# Draft Genome Sequence of *Streptomyces fradiae* ATCC 19609, a Strain Highly Sensitive to Antibiotics

**DOI:** 10.1128/genomeA.01247-14

**Published:** 2014-12-04

**Authors:** Olga B. Bekker, Ksenia M. Klimina, Aleksey A. Vatlin, Natalia V. Zakharevich, Artem S. Kasianov, Valery N. Danilenko

**Affiliations:** Vavilov Institute of General Genetics RAS, Moscow, Russia

## Abstract

We report here a sequence of the genome of the *Streptomyces fradiae* ATCC 19609 strain, initially isolated from the soil, which produces tylosin. *S. fradiae* is highly sensitive to different classes of antibiotics, compared to the sensitivities of other bacteria. We have identified 9 groups of genes directly or indirectly involved in the resistome formation.

## GENOME ANNOUNCEMENT

The concept of the “resistome” presumes that natural resistance to antibiotics is mediated by a variety of genes (1). Many pathogenic strains are drug resistant (2, 3); therefore, the identification of the genomic mechanisms of this phenomenon is clinically relevant. Bacteria of the *Streptomyces* genus are used as models to elucidate these mechanisms*. Streptomyces fradiae* strain ATCC 19609 is a soil actinobacterium (4). This species has gained interest due to its exceptional sensitivities to many classes of antibiotics, such as aminoglycosides, tetracyclines, oxazolidinones, chloramphenicol, the macrolide oligomycin, and other heterocyclic antibiotics, compared to those of the model strain *Streptomyces lividans* (5). Here, we present a draft genome sequence of *S. fradiae* strain ATCC 19609, in which we have identified genes directly or indirectly involved in resistome formation. These include genes of membrane transporters, some genes of the global regulators of transcription, translation and posttranslational modification, and other genes traditionally considered to be involved in the development of resistance and tolerance of bacteria (6–12). This analysis is pertinent to an understanding of the mechanisms of antibiotic sensitivity**.**

The genome sequencing of the *S. fradiae* ATCC 19609 strain was carried out using a whole-genome shotgun sequencing approach performed on a Roche 454 GS Junior instrument (Roche, Switzerland). A total of 362,184 reads were generated. All reads were assembled to an initial draft genome of 7,670,374 nucleotides at 21-fold coverage using the GS *de novo* Assembler (version 2.8; Roche). The resulting draft genome sequence consists of 171 contigs (155 contigs >500 bases; largest contig, 362,888 bp; overall G+C content, 72.83%). The automatic functional annotation results were obtained using the NCBI Prokaryotic Genome Annotation Pipeline (http://www.ncbi.nlm.nih.gov/genomes/static/Pipeline.html).

The ATCC 19609 genome contains 6,363 predicted genes, 3 rRNA operons, and 63 tRNAs. A total of 6,179 coding sequences (CDS), 113 pseudogenes, 5 noncoding RNAs (ncRNAs), 11 clustered regularly interspaced short palindromic repeats (CRISPRs), and 79 frameshifted genes were predicted using the NCBI Prokaryotic Genome Automatic Annotation Pipeline (PGAAP). In addition, 7 insertion sequence (IS) elements were found. The only region of the phage activity was identified in the genome sequence (PHAST) (13). Within the 14,092-bp region, seven phage-like proteins, 3 hypothetical proteins, one fiber protein phage, and 2 transposase phages were found.

The analysis revealed interesting genes encoding 9 multidrug ABC transporter ATP-binding proteins, 4 WhiB family transcriptional regulators, 10 toxin-antitoxin systems, 28 serine-threonine protein kinases, 2 aminoglycoside phosphotransferases, 2 phosphinothricin *N*-acetyltransferases, 2 β-lactamases, 2 glyoxalase/bleomycin resistance protein, puromycin *N*-acetyltransferase, streptomycin 6-phosphotransferase, chloramphenicol resistance protein, tetracenomycin C resistance and export protein, bacitracin transport ATP-binding protein BcrA, and macrolide export ATP-binding/permease protein MacB-2. The F_o_F_1_-ATP synthase contains 9 subunit genes. These results will help explore the mechanisms responsible for resistance and the sensitivity of bacteria to antibiotics and can be applied in the development of new antibiotics with a novel mechanism of action.

### Nucleotide sequence accession numbers.

This whole-genome shotgun project has been deposited at DDBJ/EMBL/GenBank under the accession no. JNAD00000000. The version described in this paper is the first version, JNAD01000000.
